# Lysine Acetyltransferase Inhibitors From Natural Sources

**DOI:** 10.3389/fphar.2020.01243

**Published:** 2020-08-12

**Authors:** Francesco Fiorentino, Antonello Mai, Dante Rotili

**Affiliations:** ^1^Department of Chemistry, University of Oxford, Oxford, United Kingdom; ^2^Department of Chemistry and Technology of Drugs, Sapienza University of Rome, Rome, Italy

**Keywords:** lysine acetyltransferases, protein acetylation, natural products, enzyme inhibitors, epigenetics

## Abstract

Acetylation of histone and non-histone protein lysine residues has been widely described as a critical modulator of several cell functions in humans. Lysine acetyltransferases (KATs) catalyse the transfer of acetyl groups on substrate proteins and are involved in multiple physiological processes such as cell signalling, metabolism, gene regulation, and apoptosis. Given the pivotal role of acetylation, the alteration of KATs enzymatic activity has been clearly linked to various cellular dysfunctions leading to several inflammatory, metabolic, neurological, and cancer diseases. Hence, the use KAT inhibitors (KATi) has been suggested as a potentially successful strategy to reverse or prevent these conditions. To date, only a few KATi have proven to be potential drug candidates, and there is still a keen interest in designing molecules showing drug-like properties from both pharmacodynamics and pharmacokinetics point of view. Increasing literature evidence has been highlighting natural compounds as a wide source of molecular scaffolds for developing therapeutic agents, including KATi. In fact, several polyphenols, catechins, quinones, and peptides obtained from natural sources (including nuts, oils, root extracts, and fungi metabolites) have been described as promising KATi. Here we summarize the features of this class of compounds, describing their modes of action, structure-activity relationships and (semi)-synthetic derivatives, with the aim of assisting the development of novel more potent, isoform selective and drug-like KATi.

## Introduction

**Figure 1:**
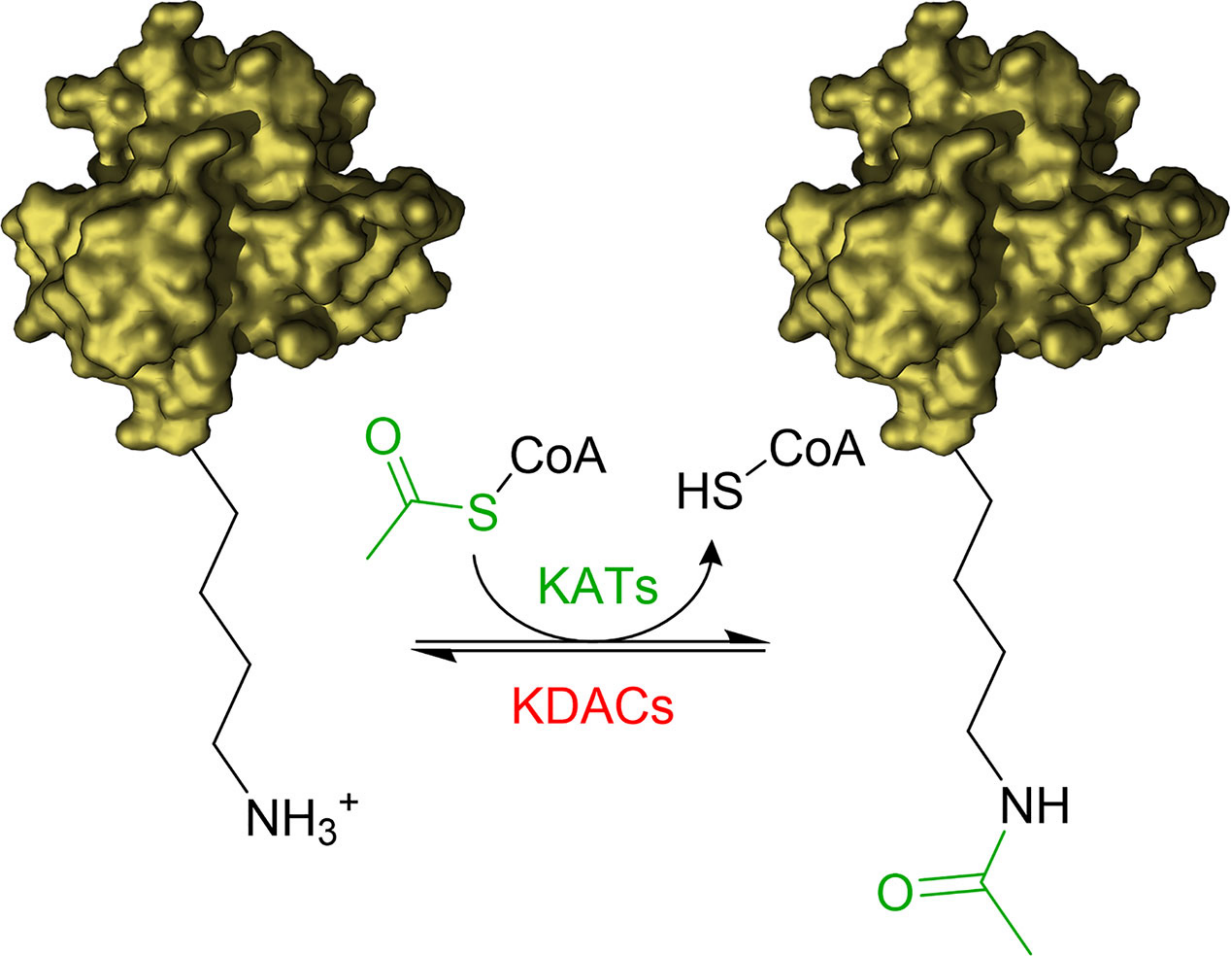
Illustration of acetylation/deacetylation equilibrium. KATs transfer an acetyl group from Acetyl-CoA to the ϵ-amino groups of lysine residues of the substrate proteins. KDACs catalyse the removal of the acetyl group.

The acetylation of the ϵ-amino groups of lysine residues is one of the most common post translation modifications (PTMs) occurring at cellular level to influence the protein function. The equilibrium between transfer and removal of acetyl groups is regulated by two classes of enzymes: lysine acetyltransferases (KATs) and lysine deacetylases (KDACs) (Falkenberg and Johnstone, 2014; Carafa et al., 2016; Fiorentino et al., 2018). KATs catalyse the transfer of an acetyl group from the co-substrate acetyl-coenzyme A (Ac-CoA) to selected lysine residues of the substrate proteins, while KDACs catalyse the hydrolysis of the acetyl group (**Figure 1**).

The targets of the acetylation/deacetylation reaction include different classes of proteins such as enzymes (for instance kinases), transcription factors, and histones (Falkenberg and Johnstone, 2014; Carafa et al., 2016; Fiorentino et al., 2018). Recent mass spectrometric studies have shown that proteins undergoing acetylation are present in approximately all subcellular compartments (Choudhary et al., 2014). Therefore, it is not surprising that acetylation is virtually related to every cellular function, ranging from signal transduction to metabolism and cell cycle regulation (Ali et al., 2018).

Acetylation prevents positive charges from forming on the amino group, thus having a significant impact on the electrostatic properties of the protein and influencing protein-protein interaction networks, as well as the cellular localisation and the sensitivity to degradation. In the case of histones, the neutralization of the positive charge of lysine residues weakens their interaction with the negatively charged DNA, thus causing chromatin decompaction and facilitating transcription (Smith and Denu, 2009). Acetylated lysine residues are also recognized by domains present in “reader” proteins called bromodomains, thus translating the acetylation reaction in specific downstream signals (Filippakopoulos and Knapp, 2014).

## KAT Families and Mechanistic Features

KAT enzymes have been arranged into three major families based on their homology to yeast orthologues, as well as mechanism of catalysis. The families comprise: the p300/CREB-binding proteins (p300/CBP); the GCN5-related *N*-acetyltransferases (GNAT), and the MOZ, Ybf2, Sas2, and Tip60 (MYST) family. Acetyltransferase activity has also been found in further proteins not classified in any of the major families, such as the transcription factor complexes TAF1/TBP and TFIIIC90, circadian locomotor output cycles protein kaput CLOCK (KAT13D), and nuclear receptor coactivator-1 (NCoA-1, also referred as SRC-1).

All KATs are characterised by a similar tertiary structure in the catalytic domain, consisting of an α/β fold, essential for co-substrate binding and catalysis, while the adjacent regions contribute to the determination of substrate specificity. Furthermore, KATs are often part of heteromultimeric complexes, and the interacting protein partners also play a key role in determining the target specificity and functions (Lee and Workman, 2007).

**Figure 2:**
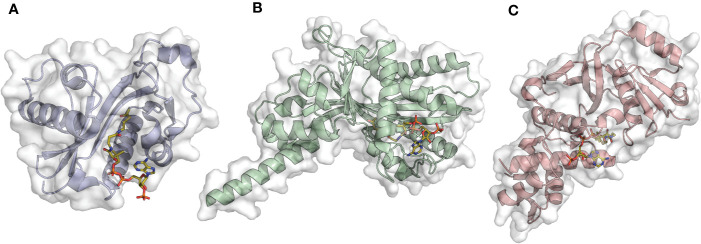
Examples of acetyltransferase domain structures from the three main KAT families in complex with the co-substrate Ac-CoA (orange sticks). **(A)** Gcn5 (PDB ID: 1Z4R); **(B)** p300 (PDB ID: 4PZS); **(C)** MOF (PDB ID: 6BA4).

The p300/CBP family comprises p300 (KAT3B, **Figure 2A**) and its paralog CBP (KAT3A). The two proteins have interchangeable functions and present high sequence and structural similarity which reflects the same mechanism of catalysis (Chan and La Thangue, 2001). The acetylation reaction follows a “hit and run” (Theorell−Chance) mechanism consisting of initial binding of acetyl-CoA to the enzyme, followed by a weak and transient interaction with the histone substrate essential for acetyl transfer. Interestingly, in this proposed mechanism the ternary complex is kinetically irrelevant for catalysis (Liu et al., 2008). Not only p300/CBP possess KAT activity, but they also have additional domains, specifically three cysteine-histidine rich domains (TAZ, PHD, and ZZ) and a bromodomain (BRD), which are critical for protein-protein interactions.

The main substrates of p300/CBP are the histone proteins H2A, H2B, H3, and H4, however, given their intricate structures, they have more than 400 interacting partners. For instance, in the context of transcriptional activation, the two KATs may act as a bridge to connect transcription factors to the transcription machinery, and acetylates histones and/or transcription factors in order to enable a transcriptional response (Dancy and Cole, 2015). Consequently, p300/CBP regulates the cellular localization and activities of a large number of proteins, particularly transcription factors (Sano and Ishii, 2001; Wang et al., 2005), thus affecting cell growth and senescence, DNA damage repair and apoptosis (Kalkhoven, 2004).

The GNAT (Gcn5 related *N*-acetyltransferase) family includes the Gcn5 (General Control Nonderepressible 5, KAT2A, **Figure 2B**) (Brownell and Allis, 1995), PCAF (p300/CBP associated factor, KAT2B) (Yang et al., 1996), the Elp3 (Elongator complex protein 3, KAT9) (Wittschieben et al., 1999), Hpa2 (KAT10), Hpa3 (Sampath et al., 2013), the mediator-complex subunit Nut1 (Lorch et al., 2000), and α-tubulin acetyltransferase 1 (α-TAT1) (Friedmann et al., 2012).

GNAT family members share four conserved 15–33 amino acids motifs indicated as A, B, C, and D, as well as various chromo- and bromodomains. The A motif contains the conserved R/Q-X-X-G-X-G/A sequence, indispensable for acetyl-CoA recognition and binding (Dyda et al., 2000). The catalytic mechanism of GNAT proteins, differently from p300/CBP, involves the formation of a ternary complex composed of the enzyme, the co-substrate acetyl-CoA, and the target protein. Following acetyl-CoA and substrate binding to the enzyme, the acetyl group is transferred to the lysine ϵ-amino group (Tanner et al., 2000).

Gcn5 and PCAF are closely related proteins, sharing 73% sequence similarity and having H3K14 as main *in vitro* substrate (Roth et al., 2001). Gcn5 acetylation activity has been shown to regulate cell cycle progression through the acetylation of CDC6 (cell division control protein 6 homolog), a critical factor for the assembly of the pre-replicative complex in G1 phase (Paolinelli et al., 2009). Similarly, PCAF is involved in cell cycle regulation, as well as transcriptional regulation and differentiation. For instance, under conditions of stress, PCAF acetylates H3 on p21 promoter, thus inhibiting cell growth (Love et al., 2012).

α-TAT1 is structurally similar to Gcn5 and acetylates Lys40 of α-tubulin (Kormendi et al., 2012; Taschner et al., 2012). Although the structural similarity to Gcn5, α-TAT1 contains a wider substrate binding pocket and further specific structural elements that play an essential role in α-tubulin specific acetylation (Friedmann et al., 2012). Given the specificity for α-tubulin, α-TAT1 activity is associated to processes such as cell adhesion, migration, and invasion (Castro-Castro et al., 2012; Aguilar et al., 2014).

The MYST family comprises the following enzymes: MOZ (monocytic leukemia zinc finger protein, KAT6A), MORF (MOZ related factor, KAT6B), MOF (males absent on the first, KAT8, **Figure 2C**), Tip60 (Tat-interacting protein, KAT5), and HBO1 (HAT bound to ORC1, KAT7). Enzymes belonging to this family possess a highly conserved catalytic region (the MYST domain) containing the acetyl-CoA binding site and zing finger motifs (Neuwald and Landsman, 1997; Sapountzi and Cote, 2011). A characteristic feature of MYST enzymes is the mechanism of catalysis consisting of a double displacement reaction (Yan et al., 2002; Wapenaar et al., 2015). In brief, the co-substrate acetyl-CoA donates the acetyl moiety to a cysteine residue, which in turn transfers it to the lysine of the substrate protein. A key role is played by a glutamate in the catalytic site that deprotonates the lysine of the substrate protein, thus facilitating the nucleophilic attack.

MOZ and MORF present high sequence homology and have the histone H3 as main target. They both form multi-protein complexes involved in signalling related to transcriptional activation (Avvakumov and Cote, 2007) and development processes such as haematopoiesis (Perez-Campo et al., 2009; Perez-Campo et al., 2013) and skeletogenesis (Crump et al., 2006).

Tip60 is the first described human member of MYST family and acetylates H4 at different Lys residues and transcription factors such as p53 (Tang et al., 2006), *c*-Myc (Patel et al., 2004), and E2F1 (Van Den Broeck et al., 2012). Given its wide range of substrates, Tip60 is involved in multiple pathways including transcriptional activation, apoptosis (Sykes et al., 2006) and DNA-damage response (Murr et al., 2006).

MOF is the latest discovered member of the MYST family and has H4K16 as main substrate, but it is also active towards H4K5, H4K8, TIP5, and p53. MOF is the acetylating subunit of two distinct complexes, MSL (male-specific lethal) and NSL (non-specific lethal), that modulate its target specificity and thus downstream effects (Ravens et al., 2014). Interestingly, evidence shows that MOF is also involved in the transcriptional regulation of mitochondrial DNA (Chatterjee et al., 2016).

As explained above, further human KATs are represented by proteins that are part of transcription factor complexes. For instance, TFIIIC90 acetylates H3 and is involved in the recruitment of RNA polymerase III for transcriptional activation (Hsieh et al., 1999).

CLOCK and NCoA-1 are co-activators of nuclear hormone receptors with substrate specificity towards H3 and H4. CLOCK is involved in circadian rhythm regulation (Doi et al., 2006), while NCoA-1- acetylation is a consequence of steroid-mediated transcriptional activation (Spencer et al., 1997).

## KATs and Diseases

Given their wide substrate specificity (including histones, transcriptional factors, kinases, and tumor suppressors) and involvement with key cellular processes, KATs play a pivotal role in cellular physiology and disease (Fiorentino et al., 2018). The dysregulation of KAT expression or enzymatic activity may lead to tumorigenesis and be the cause of diseases like inflammatory disorders, respiratory, cardiovascular, and neurological pathologies (Dekker and Haisma, 2009; Schneider et al., 2013; Huang et al., 2015; Sun et al., 2015).

For instance, p300/CBP may form chimeric proteins with the MYST family members MOZ and MOF, or the histone methyltransferase MLL which are the main cause of acute myeloid leukaemia (AML). These chimeras present aberrant catalytic activity given the presence of two active sites, thus leading to increased protein expression, particularly oncogenes (Chaffanet et al., 2000; Sun et al., 2015). Mutations and deletions of p300/CBP are related to a subset of cancer types such as lung, colon, and ovarian carcinomas (Gayther et al., 2000; Kishimoto et al., 2005), but also developmental disorders, like the Rubinstein-Taybi syndrome. This disease is characterised by mental and physical anomalies and increased susceptibility to cancer (Portela and Esteller, 2010). One of the main targets of p300/CBP is the transcription factor NF-κB. It has been shown that NF-κB acetylation is correlated to a deceased affinity for the inhibitory protein IκB, hence facilitating the expression of NF-κB target genes involved in the inflammation pathway (Schneider et al., 2013).

Gcn5 has been shown to be upregulated in a diverse range of cancer types. For instance, Gcn5 promotes the expression of cell cycle factors in non-small cell lung cancer (NSCLC) (Chen et al., 2013), as well as colon cancer, where the expression of the oncogene *c-*Myc was increased (Yin et al., 2015). Differently, PCAF may be up- or down-regulated depending on the cancer type. Its overexpression is associated with invasive urothelial carcinoma (Shiota et al., 2010), while PCAF was found to be down-regulated in oesophageal squamous cell carcinoma (Zhu et al., 2009), hepatocellular carcinoma (Li et al., 2016), ovarian and gastric cancer (Ying et al., 2010). Furthermore, Gcn5 and PCAF aberrant activity has been related to type 2 diabetes. Both proteins acetylate PGC-1α (peroxisome proliferator-activated receptor gamma co-activator 1 alpha), a co-activator of hepatic gluconeogenesis. Acetylation is a trigger for PGC-1α degradation, and an anomalous Gnc5/PCAF activity leads to a decrease in blood and hepatic glucose output (Sun et al., 2014).

The MYST family members MOZ and MORF form hybrid proteins with p300/CBP contributing to the insurgence of AML, as already discussed. Apart from p300/CBP, MOZ can also form chimeras with the nuclear receptor co-activator TIF2, and this event is also implicated in AML (Kindle et al., 2005). Similarly to PCAF, both MOF and Tip60 have tissue-specific roles in cancer. For instance, their expression is related to tumor growth in oral tongue squamous cell carcinoma (MOF) (Li et al., 2015), and prostate cancer (Tip60) (Halkidou et al., 2003). Conversely, both proteins are down-regulated in breast cancer (Pfister et al., 2008; Bassi et al., 2016). MYST family members have demonstrated to be important for neural development, indeed MORF is highly expressed in the brain and mutations are associated with neurodevelopmental disorders in humans such as the genitopatellar syndrome, a skeletal dysplasia with cerebral and genital anomalies (Campeau et al., 2012).

Given the extensive association between KATs abnormal activity and a variety of diseases, many molecules have been developed to interfere with their activity, as well as clarify their physiopathology (Fiorentino et al., 2018). To this end, many research groups have developed inhibitors of KATs (KATi) and a great amount of them are represented by natural products or their (semi)-synthetic derivatives. In recent years KATi discovery has finally succeeded in releasing highly potent and isoform selective inhibitors, along with their relative co-crystal structures. The p300 inhibitor A-485 (Lasko et al., 2017), and the MOZ/MORF inhibitors WM8014 and WM1119 (Baell et al., 2018) represent an important step forward in the field of KAT drug discovery. However, many other KAT subtypes still lack good inhibitors, which may be obtained using natural occurring products as scaffolds for focused drug design and development projects.

This review will focus on KATi obtained from natural sources describing their mechanisms of action, structure-activity relationships and (semi)-synthetic derivatives.

## KATi from Natural Sources and (Semi)-Synthetic Derivatives

The connection between anomalous protein acetylation and the onset of different diseases has inspired the research on KATi with the final goal of developing therapeutics, as well as chemical probes to further investigate KAT function.

Interestingly, over last decades, a great number of drugs and biological tools have been developed from natural products with examples including antimicrobial drugs (beta lactam and glycopeptide antibiotics), but also analgesics (aspirin and opioids) and cancer chemotherapeutics (paclitaxel and derivatives) (Dias et al., 2012; Newman and Cragg, 2012). Therefore, it is not surprising that research groups exploit natural occurring molecules to develop biologically active compounds. In fact, natural products represent a valid alternative to synthetic molecules given their higher chemical complexity and the different chemical space they occupy. Natural compounds usually present a preference of oxygen more than sulphur or nitrogen as heteroatoms, as well as a higher number of stereogenic centres and fused rings (Cherblanc et al., 2013). On the other hand, they are usually poorly selective towards their target given the presence of electrophile or redox centres and polyphenol moieties. Nevertheless, natural products have always been crucial for the development of therapeutics, representing scaffolds for the development of semi-synthetic derivatives, like in the case of the different generations of penicillin derivatives. Moreover, natural products may be a start point for ligand-based drug discovery campaigns, where the initial lead compound may be modified through (bio)isosteric replacement or may undergo structural simplification, to remove unnecessary or undesirable substructures and modulate potency and target specificity. A classic example of structural simplification are the opioid analgesics such as levorphanol or fentanyl which represent two different degrees of simplification of the parent natural molecule morphine (Wang et al., 2019).

The above-described features also characterise natural occurring KAT modulators and, although they may show some drawbacks related to their physicochemical properties, current efforts are made towards the improvement of their activity and selectivity. In fact, their optimization into new (semi)-synthetic derivatives could represent a significant milestone towards the obtainment of novel selective and potent KATi.

### Polyphenols

**Figure 3:**
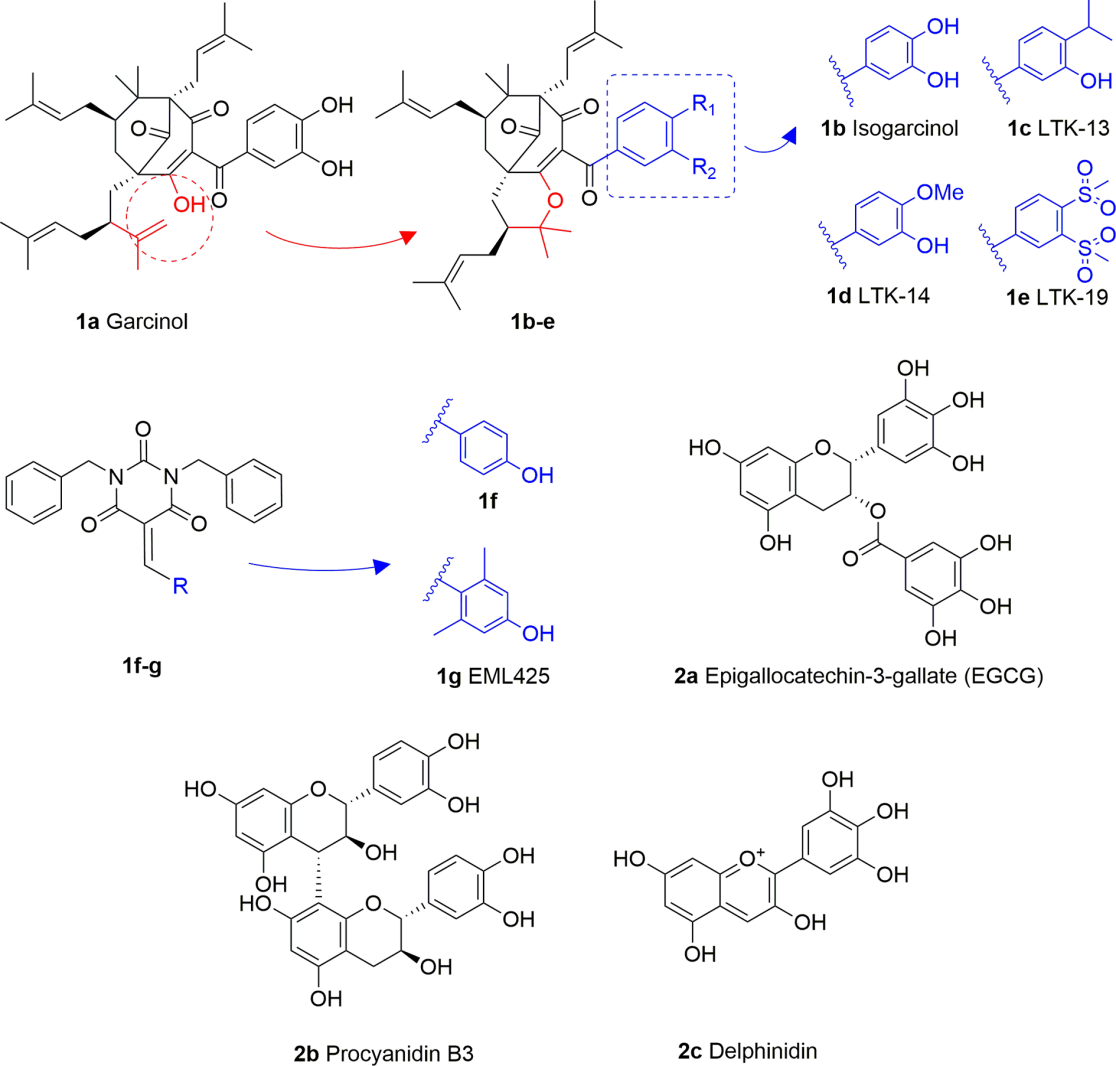
Structures and modifications of garcinol (**1a-g**), EGCG and related derivatives (**2a-b**), and delphinidin (**2c**).

Garcinol (**1A**) is a polyisoprenylated benzophenone extracted from *Garcinia indica* showing inhibitory activity in the low micromolar range towards p300 and PCAF (IC_50_ = 7 and 5 μM, respectively) (Balasubramanyam et al., 2004a). Isogarcinol (**1B**) is the result of the intramolecular cyclization of garcinol and can be extracted from *Garcinia mangostana* (Cen et al., 2013). Similarly, it shows nonspecific KATi activity towards p300 and PCAF in the same micromolar range (Mantelingu et al., 2007b). Different derivatives of **1B** were synthesized in order to improve selectivity and potency, and to decrease toxicity: LTK-13 (**1C**) and LTK-14 (**1D**) are monoaliphatic substituted derivatives, while LTK-19 (**1E**) is a bis methyl sulfonyl derivative (**Figure 3**). The three compounds showed specific inhibition of p300 with IC_50_ values in the 5–7 μM range. Among these, **1D** displayed higher efficiency *in vivo*, and impaired reproduction of HIV through the down regulation of p300-mediated acetylation of p53; moreover, it was nontoxic towards T-cells (Mantelingu et al., 2007b). Molecular simplification of **1A** led to the benzylidene barbituric acid derivative **1F** which showed selective p300 inhibition with an IC_50_ value of 2.1 μM. To overcome the susceptibly to hydrolysis of compound **1F**, two methyl groups at the *ortho* positions of the benzylidene moiety were inserted, thus leading to EML425 (**1G**), a selective inhibitor of p300/CBP active in the low micromolar range [IC_50_ (p300) = 2.9 μM and IC_50_ (CBP) = 1.1 μM]. AlphaLISA homogeneous proximity immunoassays performed by varying either histone H3 or acetyl-CoA concentration and adding increasing concentrations of **1G**, indicated a non-competitive mode of action. Despite the presence of an α,β-unsaturation would make **1G** a good Michael acceptor with potential pleiotropic mechanisms of action, remarkably, tests in human leukaemia U937 cells showed a **1G-**promoted reduction of H4K5 and H3K9 acetylation levels, and the induction of cell cycle arrest in the G0/G1 phase (Milite et al., 2015).

Epigallocatechin-3-gallate (EGCG, **2A**), is a non-selective KATi active against p300, CBP, PCAF and Tip60 with IC_50_ values of 30, 50, 60, and 70 μM, respectively (Choi et al., 2009). However, polyphenols like **2A** are able to interact with an extensive range of proteins and the optimization of their activity for every single target is quite challenging (Mai et al., 2008). Procyanidin B3 (**2B**, **Figure 3**) showed up to 90% KAT activity inhibition in a dose-dependent fashion, as well as 60% p300 activity following a non-competitive mode of action. In addition, it inhibited p300-mediated androgen receptor acetylation and acetylation-dependent prostate cancer cell proliferation (Choi et al., 2011).

Delphinidin (**2C**, **Figure 3**), is an anthocyanidin extracted from pomegranate (*Punica granatum*), showing specific p300/CBP inhibition (IC_50_ = 30 μM). This compound has shown potential as anti-inflammatory agent as it showed suppression of inflammatory cytokines expression in MH7A cell through hypoacetylation of NF-κB, as well as inhibition of cytokine release in Jurkat T lymphocyte cell lines (Seong et al., 2011).

**Figure 4:**
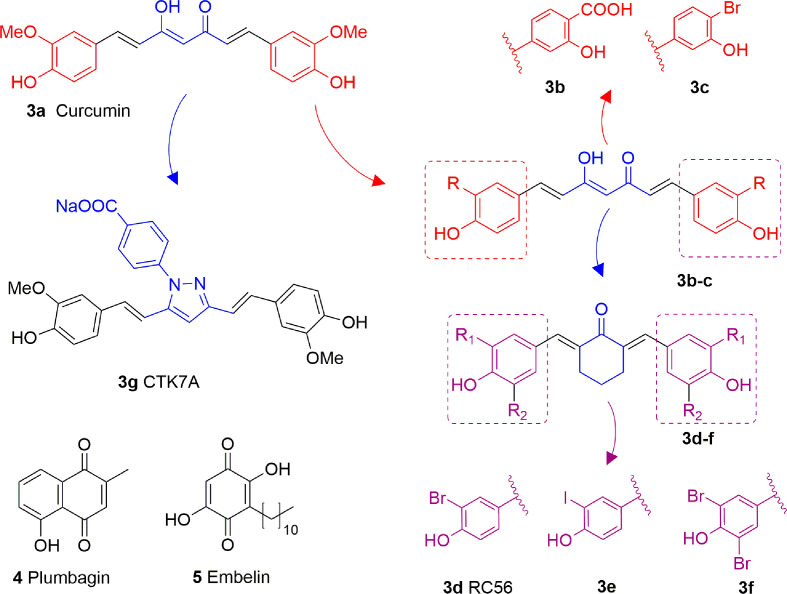
Structures and modifications of curcumin and its derivatives (**3a-g**), plumbagin (**4**) and embelin (**5**).

Curcumin (**3A**) is a natural product extracted from the rhizome of turmeric (*Curcuma longa)*, a common preparation used in both traditional Indian and Chinese medicines. Curcumin is a non-competitive p300 inhibitor (IC_50_ = 25 μM) (Balasubramanyam et al., 2004b) presenting a mode of action likely relying on the covalent binding to the target enzyme through its cinnamoyl moieties serving as Michael acceptors (Marcu et al., 2006). Interestingly, curcumin induced down-regulation of mGlu2 receptor in mouse spinal cord after systemic administration and decreased the acetylation levels of histones H3 and H4 in dorsal root ganglia (DRG) (Zammataro et al., 2014). On the other hand, given its covalent mode of action and its polyphenolic nature, curcumin is not a selective KATi and several additional targets have been identified so far, including COX2, protein kinase C, thioredoxin reductase, tubulin, and 5-lipoxygenase (Anand et al., 2008). Because of its pleiotropic nature, despite numerous studies indicated the positive effects of curcumin in various pathologies such as diabetes (Maradana et al., 2013), cardiovascular diseases (Srivastava and Mehta, 2009) and others, it is hard to attribute them to KAT inhibition. Curcumin was also shown to have membrane disruption properties, thus that some of its cellular effects may be the result of this additional feature (Ingólfsson et al., 2014). Curcumin has been evaluated in more than 120 clinical trials for various conditions, however the outcomes of the completed double blinded, placebo controlled studies (including trials for conditions such as pancreatic and colon cancer or Alzheimer’s disease) have highlighted the *in vivo* inefficacy of curcumin. Indeed, features like chemical instability, unspecific cross-reactivity, and low oral bioavailability hampered the translation of *in vitro activity* into *in vivo* effects (Nelson et al., 2017). However, in order to improve its potency and selectivity, many research groups developed several synthetic derivatives of curcumin such as compounds **3B** and **3C**, where the methoxy residues were replaced by a carboxylic acid or a bromine atom, respectively (**Figure 4**). Both derivatives showed improved inhibitory activity towards p300, in fact the IC_50_ values were 33 and 21 μM, respectively, while curcumin showed an IC_50_ value higher than 400 μM in the same biophysical assay. Compound **3D** (RC56) is a cyclic ketone derivative of **3C** showing improved potency towards p300 (IC_50_ = 5 μM) and represents the scaffold for the development of various analogues, including **3E** and **3F**, the iodinated and dibrominated derivatives of **3D**, respectively (**Figure 4**). While the iodinated analogue **3E** displayed slightly lower potency (IC_50_ = 8.1 μM), the dibrominated derivative **3F** had an increased p300 inhibitory activity (IC_50_ = 2.3 μM) (Costi et al., 2007; Mai et al., 2008). These data suggest that the presence of electron-withdrawing substituents in the phenyl ring is favourable for protein-ligand interactions. Finally, the sodium salt of phenylpyrazolocurcumin CTK7A (**3G**, **Figure 4**) is a water-soluble inhibitor of p300 and PCAF displaying a mixed type p300 inhibition for acetyl-CoA and histones. It suppressed H3 acetylation in KB cells (oral squamous cell carcinoma), being more active towards H3K14 than H3K9 and inhibited tumor proliferation in a related mouse xenograft model (Arif et al., 2010).

### Quinones

Plumbagin (**4**, **Figure 4**) is a hydroxynaphthoquinone extracted from *Plumbago rosea* roots, which has been shown to inhibit KAT activity *in vitro* and in cells with IC_50_ values for p300 and PCAF of 20 and 50 μM, respectively, with a non-competitive mode of action (Vasudevarao et al., 2014). Docking investigations combined with site-directed mutagenesis of p300 catalytic domain suggested that the hydroxyl group of **4** forms a crucial hydrogen bond with Lys1358 in the active site of the enzyme, thus it is crucial for inhibition, as confirmed by the loss of inhibitory activity of derivatives where the hydroxyl group was substituted with other moieties (Ravindra et al., 2009). Compound **4** suffers from high thiol reactivity due to the presence of a Michael acceptor, which can be overcome through methylation on C3, still retaining the KAT non-competitive inhibition (Vasudevarao et al., 2014).

Embelin (**5**, **Figure 4**) is a hydroxybenzoquinone derivative obtained from *Embelia ribes* berries. It displayed H3K9 acetylation suppression in mouse models and PCAF *in vitro* inhibitory activity (IC_50_ = 7.2 μM). Molecular modelling studies have postulated that the hydroxyl group and the undecyl chain are necessary for ligand-protein interaction. Indeed, the hydroxyl group interacts with the amide backbone through hydrogen bonds, while the alkyl chain is inserted into a narrow hydrophobic groove near the binding site of the β-mercaptoethylamine portion of CoA. Experimental works further confirmed these findings as the conversion of one hydroxyl group to methylamine or the shortening of the alkyl chain to a 10-carbon tail completely eliminates PCAF binding (Modak et al., 2013).

### Anacardic Acid and Derivatives

Anacardic acid (**6A**) is a 6-pentadecylsalicylic acid extracted from the cashew nut shells and has been indicated as a non-competitive and non-selective inhibitor of KAT enzymes with a broad range of IC_50_ values, depending on the reports. Indeed, IC_50_ values span from 8.5 μM (Balasubramanyam et al., 2003) and over 1,000 μM (Wu et al., 2009) in the case of p300, and from 5 μM (Balasubramanyam et al., 2003) to 667.1 μM (Wu et al., 2009) in the case of PCAF. Anacardic acid also showed inhibitory activity towards MYST family members, in particular Tip60 (IC_50_s from 64 to 347.6 μM) (Wu et al., 2009; Ghizzoni et al., 2012) and MOF (K_i_ and IC_50_ values of 64 and 43 μM, respectively) (Ghizzoni et al., 2012; Wapenaar et al., 2015). At cellular level, administration of **6A** induced suppression of NF-κB signalling as a consequence of the inhibition of the acetylation of the p65 subunit (Sung et al., 2008). However, similarly to curcumin, **6A** inhibits multiple proteins (Hemshekhar et al., 2012), thus its cellular effect might be a consequence of interactions with targets other than KAT enzymes. Furthermore, **6A** presents low cell permeability, thus different derivatives have been synthesized to reduce the lipophilicity, as well as to improve the inhibitory potency and isoform selectivity.

**Figure 5:**
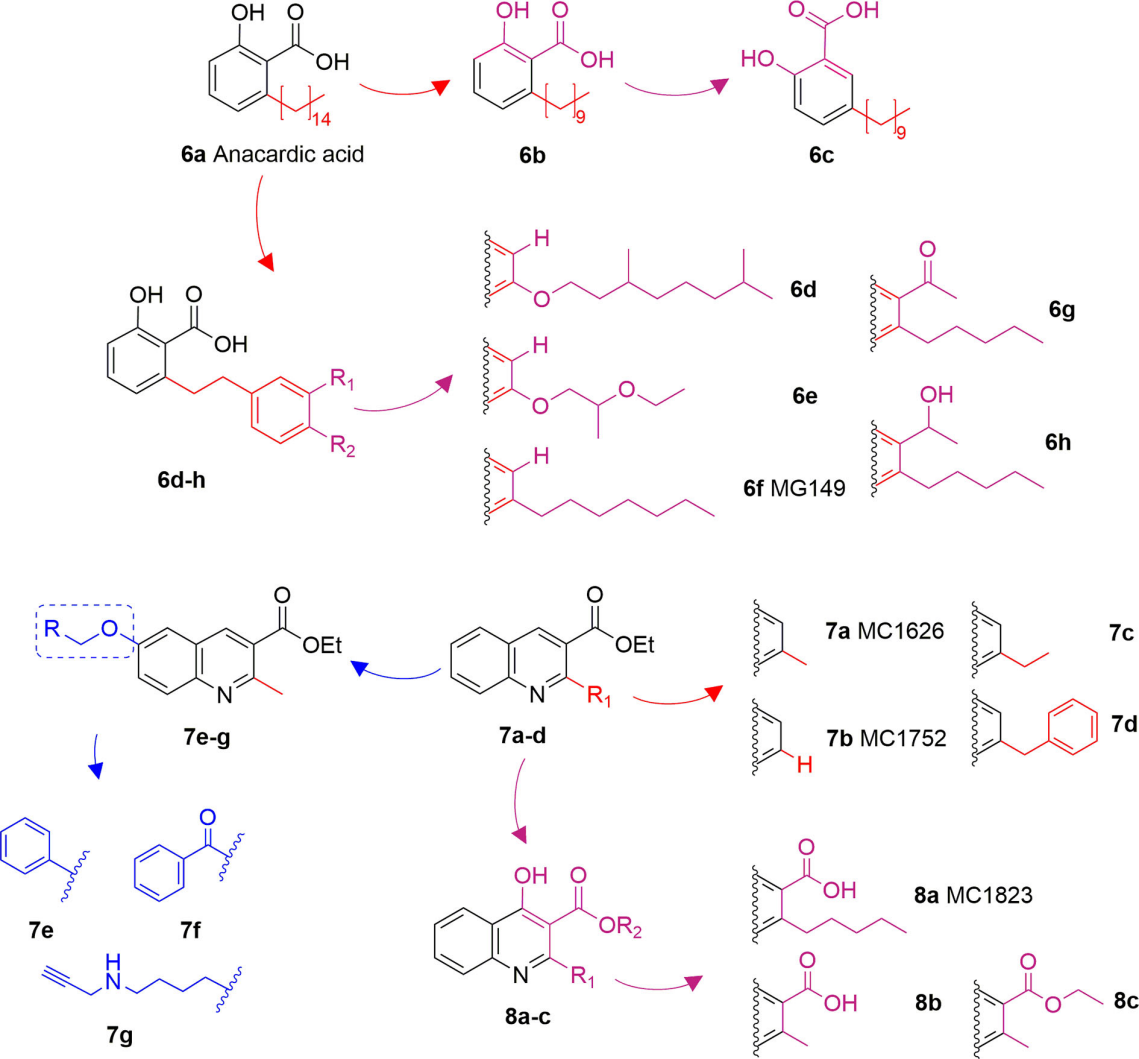
Structures and modifications of anacardic acid and its derivatives (**6a-h**, **7a-g**, and **8a-c**).

Changes in the alkyl chain length and regiochemistry led to compounds **6B** and **6C**, which exhibited inhibition towards MOF (IC_50_ = 37 and 57 μM, respectively). Compound **6B** possesses a decyl aliphatic chain instead of the pentadecyl chain of **6A** and, while **6C** is a **6B** derivative where the alkyl chain position has been switched from *ortho* to *meta* with respect to the carboxyl group (**Figure 5**). Furthermore, derivatives bearing shorter alkyl chain lengths completely lost inhibitory activity, indicating the importance of the hydrophobic contacts played by the aliphatic chain for the ligand-protein interactions (Wapenaar et al., 2015). Replacement of the alkyl chain at 6-position with a substituted phenethyl moiety led to compounds **6D-6H** (**Figure 5**), which were tested against p300, PCAF, and Tip60, displaying more than 75% inhibition of Tip60 at 200 μM (Ghizzoni et al., 2010; Ghizzoni et al., 2012). Amongst them, derivative **6F** (MG149) was the most potent [IC_50_ (Tip60) = 74 μM, IC_50_ (MOF) = 47 μM], showing competitive inhibition towards acetyl-CoA in Tip60 and uncompetitive inhibition towards acetyl-CoA in MOF (Ghizzoni et al., 2012). Furthermore, it displayed KAT inhibitory activity in cell based assays where the acetyltransferase activity was measured in hippocampus, amygdala and prefrontal cortex nuclear extracts (Ghizzoni et al., 2012), and inhibited the expression of pro-inflammatory genes in murine precision-cut lung slices (van den Bosch et al., 2017).

Compounds **7A** (MC1626) and **7B** (MC1752) can be regarded as quinoline analogues of anacardic acid that were identified through a phenotypic screening in *S. cerevisiae*. Indeed, both molecules impaired yeast cell growth resembling the effects of Gcn5 deletion mutants. In particular, **7A** also suppressed acetylation and gene transcription mediated by Gcn5 (Ornaghi et al., 2005). To improve inhibitory activity, novel **7A** derivatives presenting alternative aliphatic/aromatic chains at the C2-quinoline or additional residues at the C6-quinoline positions have been designed and synthesized. In compounds **7C** and **7D** the 2-methyl group is replaced by *n*-propyl (**7C**) or benzyl (**7D**) groups, while molecules **7E-G** are derivatives of **7A** bearing side chains at C6 (**Figure 5**). These changes improved p300 inhibitory activity and selectivity compared to **7A**. Indeed, whilst **7A** presented 22.6% p300 inhibition when tested at 100 μM, **7E-G** showed 58.5–69.3% of p300 inhibition when evaluated at the same concentration. Among the three compounds, the C6 substituted molecule **7G** was the most potent (69.3% p300 inhibition at 100 μM, and IC_50_ = 57.5 μM). Moreover, both **7C** and **7G** caused a massive reduction in H3 and H4 acetylation levels in human leukaemia U937 cells (Lenoci et al., 2014).

Further quinoline analogues of **6A** are compounds **8A** (MC1823), **8B**, and **8C**. In this case, the three substituents of the quinoline core retain the same relative positions as in **6A**. In compound **8A** a linear pentyl tail replaces the pentadecyl chain (Mai et al., 2006), while **8B** is a 2-methyl derivative, and **8C** is the ethyl ester of **8B** (**Figure 5**). Compound **8A** displayed 81% CBP inhibition at 50 μM, while **8B** and **8C** caused more than 80% inhibition of both p300 and CBP at 50 μM (Mai et al., 2009). When compared to **6A** in a nuclear extract KAT activity inhibition assay at the concentration of 50 μM, compound **8A** decreased the total KAT activity by 30%, while **6A** by just 15% (Mai et al., 2006).

**Figure 6:**
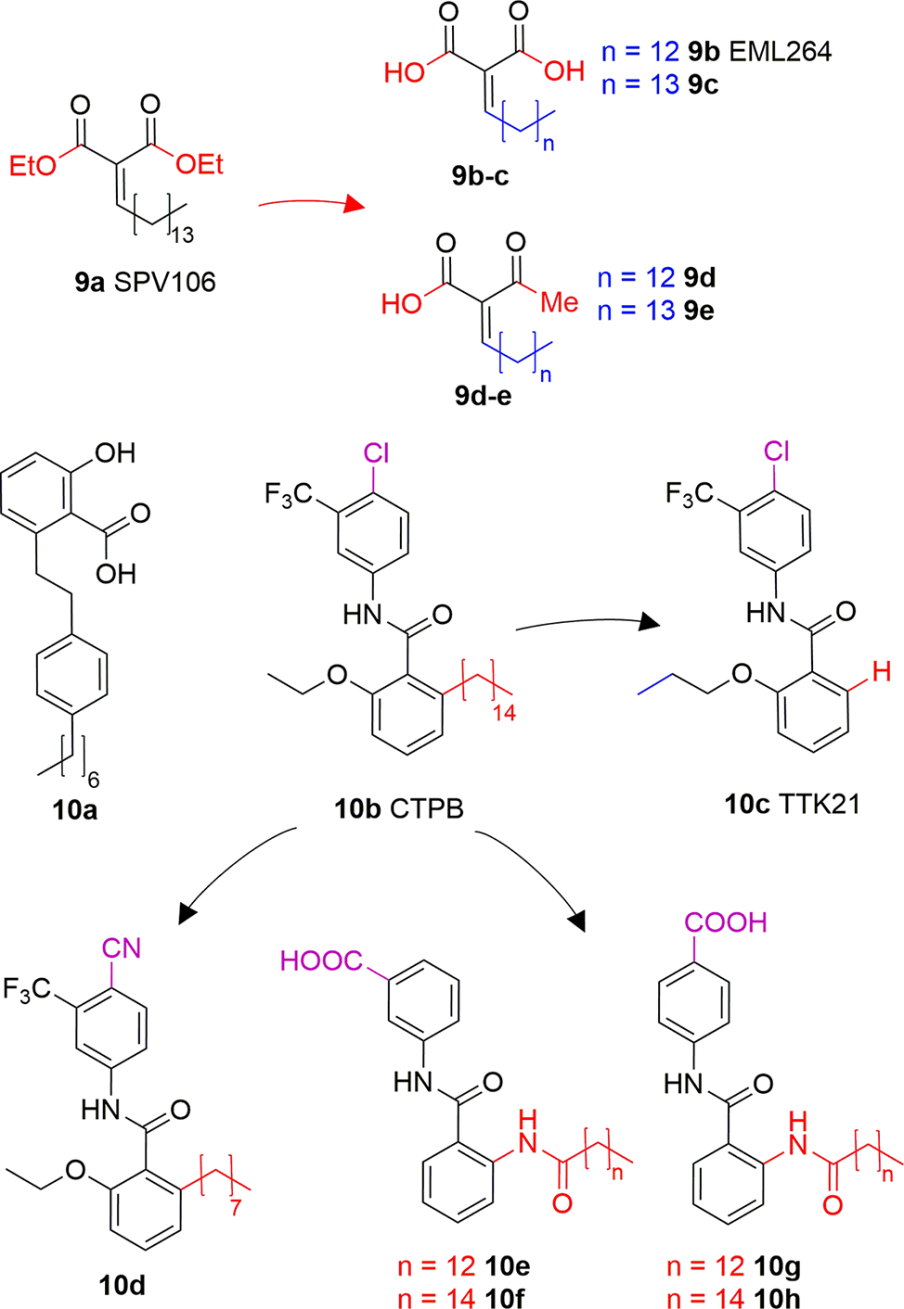
Structures and modifications of anacardic acid derivatives with mixed KAT inhibiting/activating properties: LoCAMs (**9a-e**) and compounds **10a-h**.

Long chain alkylidenmalonates (LoCAMs) are a class of KAT modulators derived from the molecular simplification of anacardic acid. SPV106 (**9A**) is the parent compound of this series showing p300/CBP inhibition comparable to **6A**, as well as a peculiar PCAF activation (Sbardella et al., 2008). SPV106 has been subjected to various modifications leading to two interesting series of compounds (**Figure 6**): bicarboxylic (**9B** and **9C**) and acetoacetic derivatives (**9D** and **9E**). Bicarboxylic derivatives are obtained from the hydrolysis of SPV106 ethyl esters and differ in the length of the aliphatic tail (14 and 15 carbons for **9B** and **9C**, respectively). They are both low micromolar p300 inhibitors (IC_50_ = 1.3 and 1.1 μM for **9B** and **9C**, respectively) and weak PCAF inhibitors. On the other hand, the acetoacetic derivatives are p300 inhibitors (IC_50_ = 2.4 and 4.7 μM for **9D** and **9E**, respectively), but also potent PCAF activators (Castellano et al., 2015). Considering the activities of LoCAM compounds towards p300 and PCAF we can conclude that the ester hydrolysis improves p300 inhibitory activity, but decreases affinity towards PCAF, whilst replacement of carboxylic moiety with an acetyl group is favourable for PCAF activation (Sbardella et al., 2008; Castellano et al., 2015). Furthermore, variations to the length of the alkyl chain or introduction of a heteroatom is detrimental for the interaction with both enzymes (Castellano et al., 2015).

Another reported anacardic acid derivative presenting both KAT inhibitory and activating properties is compound **10A** which inhibits MOF and Tip60 with IC_50_ values of 47 and 64 μM, respectively, and exhibits PCAF activation (Ghizzoni et al., 2012). Differently, the anacardic acid benzamide derivative **10B** (**Figure 6**) displays p300 activation as confirmed by the observed p300-dependent transcriptional activation (Balasubramanyam et al., 2003; Mantelingu et al., 2007a). The removal of CTPB long alkyl chain (compound **10C**, TTK21, **Figure 6**) does not impair the p300/CBP activating ability (Chatterjee et al., 2013). Remarkably, compound **10D**, presenting an octyl chain and a nitrile group replacing the chlorine has a reversed activity, showing 50% p300 inhibition at 100 μM *in vitro*. It also inhibited p300 KAT activity and induced apoptosis in immortalized HEK cells (Souto et al., 2008). Further derivatives replacing the alkyl chain with a long chain inverted amide (**10E-H**, **Figure 6**) displayed inhibitory activity against PCAF and were active in several human cancer cell lines (Park and Ma, 2012). Compound **10E** showed 79% PCAF inhibition at 100 μM, which was higher than **6A**, used as a positive control for the experiment. When translated to cytotoxic assays, IC_50_ values of compounds **10E-H** were ranging from 25 to 80 μM in HCT, A549, HT-29, Hep3B, MDA- 231, and HeLa cancer cell lines (Park and Ma, 2012).

### Alkaloids

**Figure 7:**
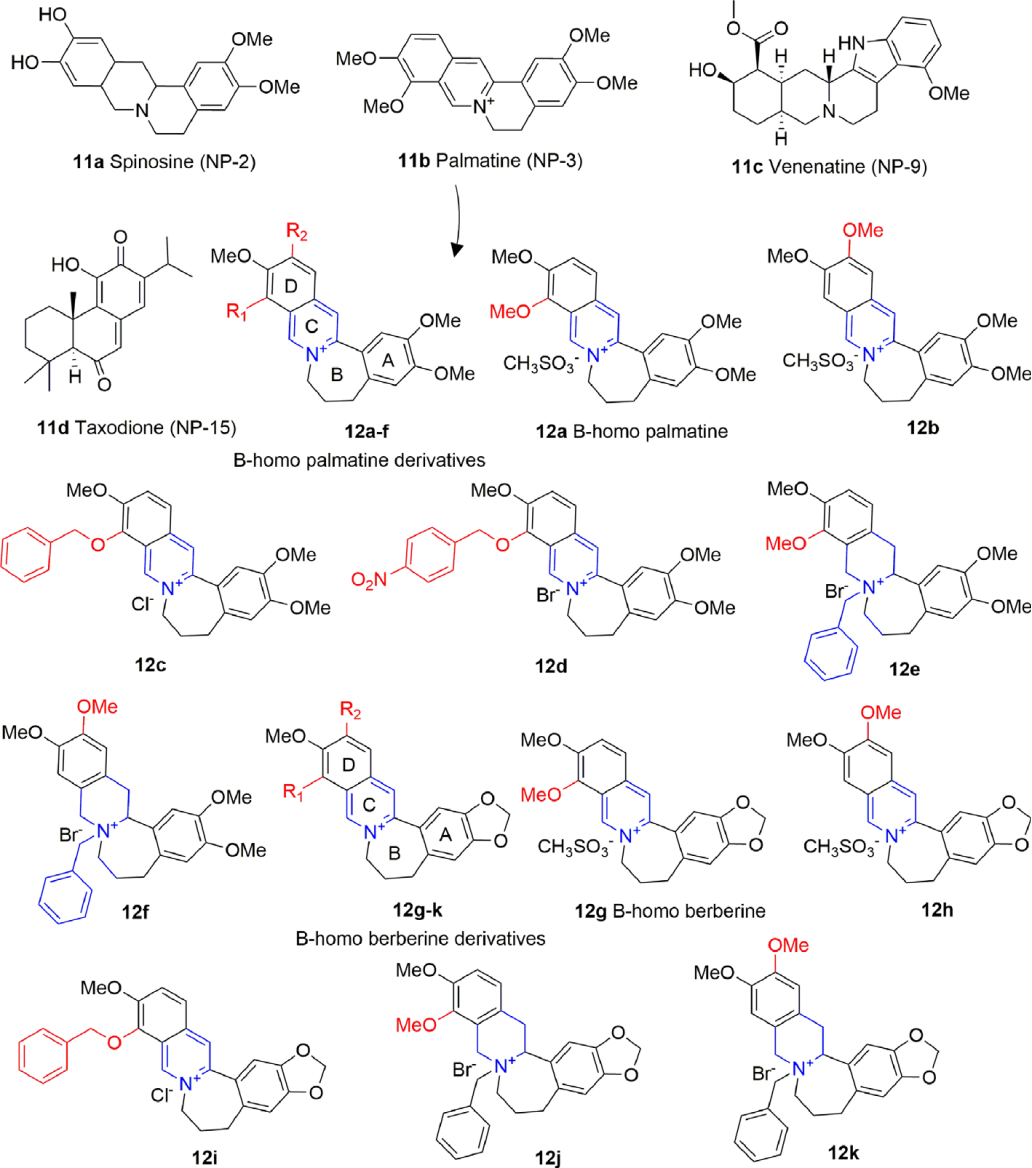
Structures and (semi)-synthetic derivatives of alkaloids with KAT inhibitory activity (**11a-d** and **12a-k**).

Alkaloids are plant-derived compounds containing multiple cycles and nitrogen atoms. A structure-based virtual screening identified four alkaloids (**11A-D**, **Figure 7**) possessing inhibitory activity against p300 in the low micromolar range, and against PCAF in the medium micromolar range [p300: IC_50_ (**11A**) = 0.69 μM, IC_50_ (**11B**) = 1.05 μM, IC_50_ (**11C**) = 0.58 μM IC_50_ (**11D**) = 4.85 μM; PCAF: IC_50_ (**11A**) = 14.13 μM, IC_50_ (**11B**) = 10.0 μM, IC_50_ (**11C**) = 27.1 μM IC_50_ (**11D**) = 7.16 μM]. When docked on p300, these compounds share a similar binding mode, consisting on the formation of hydrogen bonds with key residues Arg1410, Thr1411, and Trp1466, similarly to acetyl-CoA. However, none of these compounds showed activity on cancer cell lines, apart from **11D**, although the authors of the paper do not exclude off-target effects in this case (Guo-Bo et al., 2016).

Compound **11B** (also indicated as Palmatine) has been used as source of inspiration to design B-homo palmatine (**12A-F**) and B-homo berberine derivatives (**12G-K**) all endowed with a central dihydroazepine B ring (**Figure 7**). Amongst them, B-homo palmatine derivative **12B** showed the best potency towards p300 (IC_50_ of 0.42 μM) with a 10-fold increase compared to its parent compound **12A** (IC_50_ = 4.5 μM). Similarly, the B-homo berberine derivative **12H** showed a better inhibitory activity than its parent compound **12G** (IC_50_ = 9.2 and 1.8 μM for **12G** and **12H**, respectively). The SAR evaluation indicates that generally B-homo berberine derivatives are less active than the palmatine ones, and the shift of the methoxy group from R_1_ to R_2_ position improves the inhibitory activity. Moreover, the replacement of methoxy groups with bulky substituents such as benzyloxy moieties does not affect or even decreases the activity of these compounds against p300 [IC_50_ (**12C**) = 4.7 μM, IC_50_ (**12D**) = 9.4 μM, IC_50_ (**12I**) = 2.5 μM], and the addition of a benzyl group to the quaternary amine massively decreases the inhibitory activity in both series [IC_50_ (**12E**) = 4.7 μM, IC_50_ (**12F**) = 7.4 μM, IC_50_ (**12J**) = 34 μM, IC_50_ (**12K**) = 44 μM] (Yang et al., 2018).

### Prostaglandins

**Figure 8:**
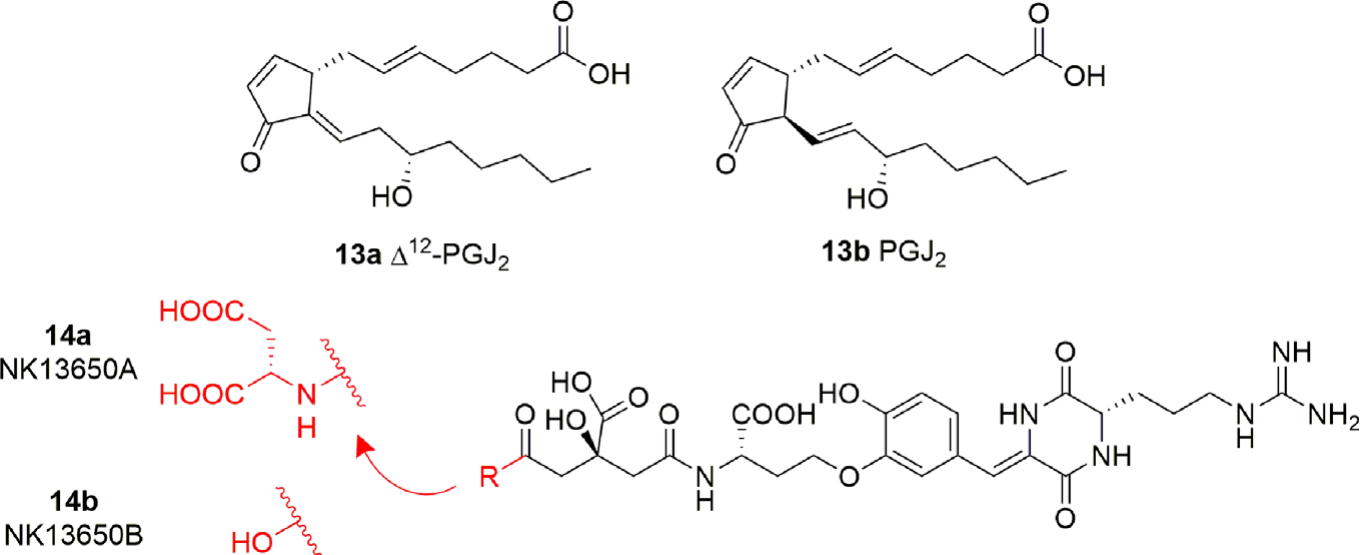
Structures of prostaglandins (CyPGs) and *Penicillium* peptide metabolites with KAT inhibitory activity (**13a-b** and **14a-b**).

Some cyclopentenone prostaglandins (CyPGs) were shown to possess p300 inhibitory activity (Ravindra et al., 2012). In particular, Δ12-PGJ_2_ (**13A**) and PGJ_2_ (**13B**) (**Figure 8**) displayed IC_50_ values of 0.75 μM and > 2 μM, respectively. Docking studies suggest that **13A** assumes a conformation such that the electrophilic carbon of the α,β-unsaturation in the cyclopentenone ring is close enough to Cys1438 of p300 to form a covalent Michael adduct. Further experiments involving site-directed mutagenesis of the p300 KAT domain, peptide competition assays, and mass spectrometric analysis validated the hypothesis of the covalent interaction of Δ12-PGJ_2_ with Cys1438. Both Δ12-PGJ_2_ and PGJ_2_ were found to inhibit H3 histone acetylation in cell-based assays. In addition, Δ12-PGJ_2_ also inhibited acetylation of the HIV-1 Tat by recombinant p300 *in vitro*. This effect was translated in U1/HIV cells (human monocytic cells chronically infected with HIV-1), contributing to the reduction of HIV viral gene expression (Ravindra et al., 2012).

### Peptides

The peptide metabolites of *Penicillium* species **14A** (NK13650A) and **14B** (NK13650B) (**Figure 8**) are potent and selective inhibitors p300 with IC_50_ values of 11 and 22 nM, respectively. Remarkably, they suppress the transcriptional activation mediated by the oestrogen and androgen receptors and reduce the cell viability in tumor cell lines, including prostate cancer cells (Tohyama et al., 2012). Their peptide nature is the main cause of low cell absorption and metabolic instability, however given their high potency and selectivity these compounds represent a good starting point for the development of optimized peptidomimetic drugs.

## Conclusions and Perspectives

Over the past decades, KAT aberrant activity has been connected to a vast range of diseases, particularly cancer. Thus, it is not surprising that great efforts have been made towards the discovery of KAT modulators. Natural compounds have always been a valid starting point for the development of small molecule drugs and, as we have described in this review, a great number of KATi have been obtained from or are indeed (semi)-synthetic derivatives of plant extracts or other natural sources (**Table 1**).

**Table 1:** Most significant KAT modulators from natural sources.

Compound	Structure	*In vitro* activity	Cell based activity	References
**1a** Garcinol	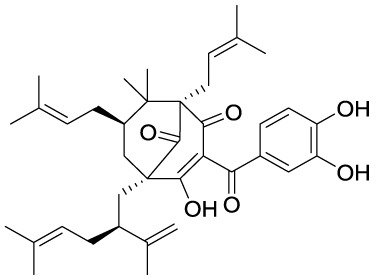	**p300** IC_50_ = 7 μM **PCAF** IC_50_ = 5 μM	Histone hypoacetylation & induction of apoptosis in various cancer cell lines.	Balasubramanyam et al., 2004a
**1d** LTK-14	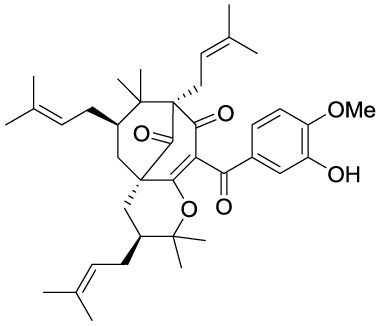	**p300** IC_50_ = 5–7 μM	*In-vivo* impairment of HIV reproduction through inhibition of p300-mediated acetylation of p53	Mantelingu et al., 2007b
**1g** EML425	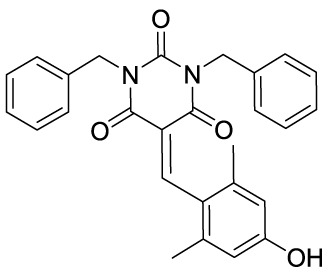	**p300** IC_50_ = 2.9 μM **CBP** IC_50_ = 1.1 μM	Decrease of H4K5 and H3K9 acetylation levels and cell cycle arrest in G0/G1 phase in human leukaemia U937 cells.	Milite et al., 2015
**2a** Epigallo-catechin-3-gallate (EGCG)	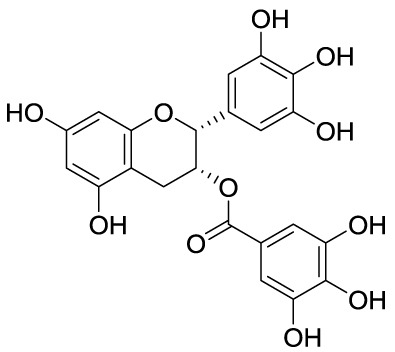	**p300** IC_50_ = 30 μM **CBP** IC_50_ = 50 μM **PCAF** IC_50_ = 60 μM **Tip60** IC_50_ = 70 μM	Disruption of NF-κB signalling.	Choi et al., 2009
**3a** Curcumin	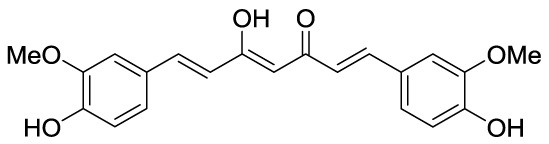	**p300** IC_50_ = 25 μM	Inhibition of p300-dependent acetylation of histone H3/H4 & p53.	Balasubramanyam et al., 2004b
**5** Embelin	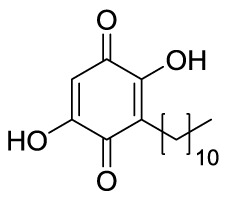	**PCAF** IC_50_ = 7.2 μM	Reduction of PCAF-mediated MyoD acetylation in HEK293T cells.	Modak et al., 2013
**6a** Anacardic acid	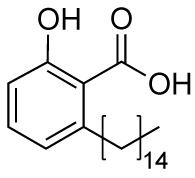	**p300/CBP** IC_50_ = 5–1,000 μM **PCAF** IC_50_ = 5–667.1 µM **Tip60** IC_50_ = 64–347.6 µM **MOF** IC_50_ = 43–64 μM	Repression of NF-κB signalling through inhibition of p65 subunit acetylation.	Wapenaar et al., 2015 Wu et al., 2009 Balasubramanyam et al., 2003 Ghizzoni et al., 2012 Hemshekhar et al., 2012
**8g**	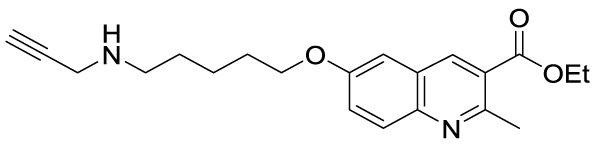	**p300** IC_50_ = 57.5 μM	Decrease in H3 & H4 acetylation in human leukaemia U937 cells.	Lenoci et al., 2014
**9a** SPV106	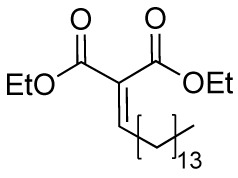	**p300/CBP** 74% inhibition @50 μM **PCAF** 137% activation @100 μM	Apoptotic effect and block of cell cycle in S phase in human leukaemia U937 cell line.	Sbardella et al., 2008 Castellano et al., 2015
**10e**	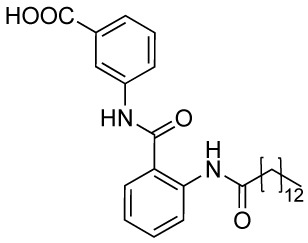	**PCAF** 79% inhibition @100 μM	Cytotoxic effect in various cancer cell lines with IC_50_ values ranging from 25 to 80 μM	Park and Ma, 2012)
**14a** NK13650A	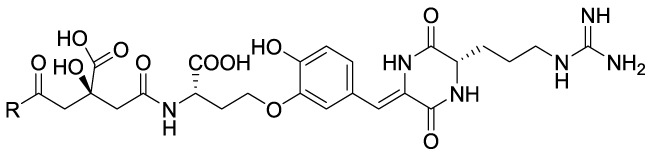 R = NH-Aspartic acid	**p300** IC_50_ = 11 nM	Repression of transcription mediated by androgen and estrogen receptors & cell viability reduction in various cancer cell lines.	Tohyama et al., 2012

However, many reported naturally derived KATi present poorly understood mechanism of action and have many off-target effects. Therefore, often further structure-activity optimization efforts are necessary to obtain the desired potency, selectivity, and in cell activity. This would be particularly beneficial not only for the obtainment of clinically active drugs, but also for the development of biological tools to further understand the intricate and multifaceted roles of the various KAT isoforms in the context of cellular homeostasis. In fact, while targeting different proteins may be useful to obtain the desired phenotypic effect (for instance, decreased expression of oncoproteins), especially in the epigenetics field (Tomaselli et al., 2020), a biological probe must have a specific target in order to correlate the observed phenotypic effect to a specific molecular interaction.

The release of the co-crystal structures of the recently discovered p300 inhibitor A-485 (Lasko et al., 2017), and the MOZ/MORF inhibitors WM8014 and WM1119 (Baell et al., 2018) have paved the way for the development of novel highly potent and selective KATi. Nonetheless, may other KAT isoforms remain untargeted and natural products represent an ideal starting point for the development of novel therapeutics using both structure-based and ligand-based drug discovery approaches.

We envisage that the impact of natural products in the development of KATi, and more in general in drug discovery, will continue to be central. The continuous improvements in protein purification, bioanalytical technologies (Johnson et al., 2011), and structural biology approaches such as cryo-electron microscopy (Poepsel et al., 2018) and structural mass spectrometry (Mehmood et al., 2015; Fiorentino et al., 2019), will further facilitate the evaluation and the employment of these molecules in research.

## Author Contributions

FF, AM, and DR contributed to conception, manuscript writing, and proof reading. FF and DR searched the literature. FF and DR prepared the figures and the table. DR supervised and coordinated the whole writing work. All authors contributed to the article and approved the submitted version.

## Funding

This paper was funded by PRIN 2016 (prot. 20152TE5PK) (AM), Ricerca Finalizzata 2013 PE-2013-02355271 (AM), AIRC 2016 (n. 19162) (AM), Progetto di Ateneo ‘Sapienza’ 2017 n. RM11715C7CA6CE53 (DR), and NIH (n. R01GM114306) (AM). FF holds a SABS CDT Studentship supported by the EPSRC and the MRC (EP/L016044/1).

## Conflict of Interest

The authors declare that the research was conducted in the absence of any commercial or financial relationships that could be construed as a potential conflict of interest.
